# Summary of Results and Discussions From the Gene-Based Tests Group at Genetic Analysis Workshop 18

**DOI:** 10.1002/gepi.21824

**Published:** 2014-08-11

**Authors:** Heather J Cordell

**Affiliations:** Institute of Genetic Medicine, Newcastle UniversityNewcastle upon Tyne, United Kingdom

**Keywords:** rare variants, burden tests, collapsing tests, SKAT

## Abstract

I present a summary of the results and discussions held within the working group on gene-based tests at Genetic Analysis Workshop 18 (GAW18). The main focus of interest in our working group was modeling the action of combinations or “groups” of genetic variants, with a group of variants most often defined as a set of single-nucleotide polymorphisms lying within a known gene. Some contributions investigated the performance of previously proposed methods (particularly rare variant collapsing or burden-type methods) for addressing this question, applied to the GAW18 data, and other contributions developed novel approaches and addressed novel questions. Most approaches were successful in detecting significant effects at *MAP4* in the simulated data. No other genetic effects were consistently detected across different analyses. Low power was noted, particularly for those methods that restricted analysis to purely the subset of unrelated individuals.

## Introduction

The Genetic Analysis Workshops are research meetings held every 2 years; they are devoted to the evaluation and comparison of statistical methods for mapping, identifying, and characterizing genetic factors involved in complex diseases. Genetic Analysis Workshop 18 (GAW18) was held October 13–17, 2012, in Stevenson, Washington. The workshop consisted of a day of group discussions (among researchers who had submitted, before the workshop, short papers summarizing their findings on a particular topic), followed by 2 days of group presentations made by each group to the wider set of GAW18 participants. Group membership was assigned thematically. Here, I summarize the results and discussion held by members of the working group on gene-based tests. Participants in this group included biologists, geneticists, computer scientists, mathematicians, and statisticians, all of whom contributed to a lively discussion of issues arising from analysis of the GAW18 data.

Our working group produced 13 papers, 11 of which were subsequently submitted and accepted for publication in the GAW18 proceedings. Table 1 lists the 13 contributions and provides summary-level information, including the data used (real or simulated, which phenotypes and simulation replicates) and the analysis approaches used. The common theme of the contributions to our working group was the desire to perform analysis (linkage or association testing) of groups of single-nucleotide polymorphisms (SNPs) simultaneously, as opposed to performing single-SNP analysis (as is most often done in genome-wide association studies). This theme was also addressed by a number of researchers in the working group on collapsing methods [Sung et al., 2014] and was also pertinent to the working group on pathway-based approaches for whole genome sequence data [Aslibekyan et al., 2014], many of whose submissions involved the initial construction of a gene-based test, followed by some procedure that grouped together sets of genes known to lie within the same biological pathway, in order to conduct a final test relating to the pathway as a whole.

**Table 1:** Summary of contributions to the working group on gene-based tests

		Real data		Simulated data				
Contribution	Related (R) or unrelated (U) individuals and sample size (*N*) used (if not full data set)	Trait	Chromosome	Trait	Chromosome	Replicates	Analysis approach	Results summary
Ayers and Cordell [2014]	R (with genomic control correction)	Mean DBP	3	Mean DBP	3	1	Penalized regression of rare and common variants	Only *MAP4* (simulated data) withstands multiple testing.
Feng and Zhu [2014]	R (correction not necessary)	Unaffected for “ever HT”	All				TDT-like test for rare variants	Rare variant Q-Q plots inflated due to genotyping errors and linkage disequilibrium created by population admixture.
Gagliano et al. [unpublished data]	U (*N* = 96)	HT1	3				SKAT-O with or without up-weighting SNPs by biological function	Nothing withstands multiple testing.
Johnston and Carvalho [2014]	U (*N* = 132)	SBP1	3				Bayesian hierarchical modeling with latent genotypes	No genes showed high posterior probability of association.
Liu and Beyene [2014]	U (*N* = 129)	SBP1, DBP1, MAP1	3	SBP1, DBP1, MAP1	3	1, 1–200	TOW and VW-TOW, LASSO, and SPLS	Low power, but some findings with *P* < 0.001.
Liu et al. [2014]	R (no correction)			SBP, DBP (clustered)	3	1–200	SKAT and novel dual clustering approach	Low power (highest power of 7.5% for *MAP4*).
Mathew et al. [2014]	R (correction not necessary)	HT1	All	HT1	3	1–200	Various methods for analysis of sequence data in families	Low power, but 12 genes showing association in real data overlapped with previous genome-wide association findings.
Mukhopadhyay [unpublished data]	U (*N* = 139)	SBP and DBP (multivariate)	All				Novel method based on genotype and phenotype similarities (using all markers in a small genomic region)	275 genes showed *P* < 0.00001.
Peralta et al. [2014]	R (correction not necessary)			SBP1, Q1	All	1	Novel variance components approach	Only *MAP4* withstands multiple testing.
Wang and Wei [2014]	U (*N* = 103)	Ever HT	All				Various rare-variant tests (qMSAT, C-alpha, CMC)	*SETX* on chromosome 9 showed *P* < 5 × 10^−6^.
Yang et al. [2014]	U (*N* = 142)			SBP1	3	1–200	SKAT and GOFT	Low power, nothing withstands multiple testing.
Zhang and Lin [2014]	R (correction not necessary)	HT1	All				Novel method (PPATu) to detect imprinting	Nothing withstands multiple testing, but PPATu is more powerful than PPAT.
Zhang et al. [2014]	R (correction not necessary)			SBP1	3	1, 1–200	Various noncollapsing rare variant tests	Low power, apart from *MAP4*, which was detected in all replicates.

CMC, combined multivariate and collapsing method; DBP, diastolic blood pressure (DBP1 is DBP at time point 1); GOFT, goodness-of-fit test; HT, hypertensive (HT1 is hypertension at time point 1); LASSO, least absolute shrinkage and selection operator; PPAT, pedigree parental-asymmetry test; Q-Q, quantile-quantile; qMSAT, quality-weighted multivariate score association test; SBP, systolic blood pressure (SBP1 is SBP at time point 1); SKAT, sequence-based kernel association test; SPLS, sparse partial least squares; TDT, transmission disequilibrium test; TOW, test for testing the effect of an optimally weighted combination of variants; VW-TOW, variable weight test for testing the effect of an optimally weighted combination of variants.

The members of our working group most often defined a “group” of SNPs as the set of SNPs lying within a known gene (i.e., between the minimum transcription start position and the maximum transcription end position, as specified, e.g., in the UCSC genome browser), but they also considered other groupings based on location (genomic region), including sliding windows and intergenic regions. Group members also discussed how best to define a gene (e.g., on the basis of transcription start and end points, whether to allow some distance around these start and end points) and whether grouping by gene was too limiting a strategy, given that the ENCODE Project has shown that 80.4% of the genome has relevant biochemical function, the vast majority of which does not, in fact, lie within genes [ENCODE Project Consortium et al., 2012]. No firm conclusion was reached on these questions, but they were highlighted as important issues for further consideration.

With respect to the actual construction of the gene-based (or other grouping) test, many investigators compared the results from several different analysis approaches, the most popular being the sequence-based kernel association test, SKAT [Wu et al., 2011], or one of its successors, such as SKAT-O [Lee et al., 2012] or famSKAT [Chen et al., 2013]. During the workshop, group members carried out comparisons of the same method across different contributions and comparisons of different methods. Several contributors developed new methods or proposed improvements to existing methods, often comparing the resulting methods with existing methods, such as SKAT or SKAT-O. In addition to methodological commonalities between different contributions (described in more detail later), other common issues included whether or how to account for family relationships between individuals, whether or how to account for population stratification or population structure, whether or how to choose weights for up-weighting particular variants (e.g., on the basis of known or hypothesized biological function or minor allele frequency), and how to choose thresholds (if required) for defining a variant as being rare.

## Methods

### Methodological Commonalities

The methods used in the 13 contributions to our working group were surprisingly diverse. Liu et al. [2014] and Mukhopadhyay [unpublished data] considered methods that involved constructing multivariate phenotype similarity measures and relating these measures to genotype similarity measures. The advantage of this approach is that it allows the consideration of phenotypes measured longitudinally at all time points, as opposed to just considering a single time point (e.g., SBP1, systolic blood pressure measured at time point 1) or simply taking the mean phenotype over available time points [as was done by Ayers and Cordell 2014]. Mukhopadhyay [unpublished data] also included the two blood pressure phenotypes, SBP and diastolic blood pressure (DBP), simultaneously, allowing for their estimated covariance. Differences between the two contributions involved the precise choices of genotypic and phenotypic “similarity” used and whether any up-weighting of rare variants was performed.

Ayers and Cordell [2014] and Johnston and Carvalho [2014] developed methods that allowed effects at multiple genes or SNPs to be modeled simultaneously by means of either penalized regression or Bayesian hierarchical modeling. An attractive feature of these approaches is that they in principle allow borrowing of information across rare and common SNPs and allow the up-weighting of certain features (e.g., up-weighting SNPs according to membership within a gene or giving higher weights to rarer variants).

Feng and Zhu [2014] and Zhang and Lin [2014] considered TDT-type (transmission disequilibrium test) methods [Spielman et al., 1993] that focused on transmission of variants from heterozygous parents to affected (and in some cases unaffected) offspring. Whereas Feng and Zhu investigated the inflation in the false-positive rate, which they attributed to linkage disequilibrium created by population admixture (and possibly resulting from other factors, such as genotyping errors), Zhang and Lin tested for imprinting or a parent-of-origin bias in transmission.

#### Accounting for Population Stratification

Most contributors did not explicitly address the issue of correcting or accounting for population stratification in their analyses, although a few [e.g., Peralta et al. 2014; Wang and Wei 2014; Zhang et al. 2014] incorporated into their analyses the top five or 10 principal component scores from a principal components analysis (based generally on the genome-wide association data from unrelated individuals, with loadings projected if necessary onto the full set of individuals) as covariates. Ayers and Cordell [2014] used a genomic control correction approach [Devlin and Roeder, 1999] to adjust their significance levels (which were, as expected, inflated because of either unmodeled relatedness or population substructure). Family-based approaches, such as the TDT-like approaches implemented by Feng and Zhu [2014] and Zhang and Lin [2014] or the variance-components linkage-like approach implemented by Peralta et al. [2014], should not, in principle, be affected by population stratification. However, Feng and Zhu did attribute the inflation in positive (most likely false-positive) signals seen in their method to linkage disequilibrium between rare variants caused by population admixture, specifically admixture with populations of African ancestry, because the two genes that showed an excess of rare variants on transmitted compared to nontransmitted haplotypes contained a large number of variants present only in African samples (according to the 1000 Genomes Project database).

#### Accounting for Family Relationships

Members of our working group took various approaches to account for the family relationships between individuals in the GAW18 data set (which consisted of 464 sequenced individuals taken from 16 large pedigrees, or 959 genotyped individuals taken from 20 large pedigrees, 849 of whom had accompanying phenotype measurements available). One approach (taken, e.g., by Liu et al. [2014]) was to use all the individuals but to treat them as though they were unrelated, acknowledging that this approach would most likely produce results with an inflated false-positive rate; nevertheless, these results could still be used to compare true- and false-positive rates (when applied to the simulated data where the answers are “known”). An alternative approach, taken by Ayers and Cordell [2014], was to use all the individuals without correction and adjust (deflate) the resulting inflated test statistics by using genomic control [Devlin and Roeder, 1999].

Several contributors used methods that were specifically designed to account for family structure. Peralta et al. [2014] developed a variance-components linkage approach in which relatedness was accounted for through a kinship matrix based on known (theoretical) kinships, with an empirically estimated local (gene-specific) kinship matrix (calculated on the basis of SNP data from within the gene region) used to construct a regional test of linkage within a variance-components framework. Zhang et al. [2014] also used a variance-components and linear mixed-model framework; they accounted for relatedness using a kinship matrix based on known kinships and constructed an SNP-based test of association. The *P*-values from the test were subsequently incorporated into various noncollapsing procedures for converting rare variant (SNP-based) *P*-values into gene-based *P*-values. Zhang et al. [2014] also performed a separate analysis using famSKAT [Chen et al., 2013], an extended version of SKAT specifically designed for analysis of family data, whereas Mathew et al. [2014] used several alternative sequence-based association methods specifically designed for family data.

Feng and Zhu [2014] and Zhang and Lin [2014] considered TDT-type methods [Spielman et al., 1993] that focused on transmission of variants from parents to offspring. Although these methods could in some sense be considered to be specifically designed for family data, the fact that the GAW18 data are not nuclear family data (but rather derived from 16 or 20 large pedigrees) raises the issue of whether any correction needs to be made to account for the trios (two parents and a child) selected from a large pedigree not being themselves independent. Feng and Zhu addressed this issue by selecting a single trio from each pedigree, resulting in 15 trios for their final analysis (because one sequenced pedigree did not have any trios with sequencing data available). Although this strategy avoids having to deal with the nonindependence between trios, it results in an extremely small final data set. Zhang and Lin used all trios with complete data within each pedigree, accounting for the correlated information in the estimated variance when they computed their test statistic, and they used computer simulations (based on the true pedigree structure) to show that their resulting type I error rate was well controlled.

By far the most common strategy used by the members of our working group was to select a subset of unrelated individuals for analysis. This allowed the use of analysis methods (and software packages) that had been previously developed for analysis of unrelated individuals, and it avoided the issue of relatedness between individuals when developing any new method. The resulting sample size varied between 96 and 142 individuals (see Table 1), depending on which phenotypes were considered and what quality control procedures (resulting in various sample exclusions) were performed. The disadvantage of this strategy was the low power that one would expect as a result of the small sample size. For genome-wide association studies sample sizes in the thousands, if not tens of thousands, are required to replicably identify genetic effects operating in complex diseases. In sequencing studies, the hope is that the operating effect sizes may be larger, but nevertheless Kiezun et al. [2012] suggested that, even for sequencing studies, many thousands of individuals will be required to achieve acceptable statistical power.

#### Up-Weighting of Specific Genetic Variants

Evolutionary arguments [Gorlov et al., 2011] suggest that rare SNPs are more likely than common SNPs to be functional and to have stronger effect sizes. This observation motivated several group members to up-weight rare variants, for example, according to the inverse of the minor allele frequency [Madsen and Browning, 2009] when constructing gene-based tests. Several contributors explored the issue of up-weighting certain variants; for example, Gagliano et al. [unpublished data] up-weighted nonsynonymous SNPs and those SNPs residing in DNase I hypersensitive sites, and Liu and Beyene [2014] up-weighted rare variants, regardless of their direction of effect. Most contributors used the default weighting scheme for the method (e.g., SKAT) employed or else imposed a weighting scheme that effectively up-weighted rare variants. The threshold for defining a variant as rare was thought to be an important but often somewhat arbitrarily chosen parameter, although data-driven approaches for choosing the threshold have been proposed [Price et al., 2010]. Group members thought that investigation of such strategies and the development of alternative and optimal weighting schemes were important areas for future investigation.

## Results and Discussion

Given the small sample size (96–142 unrelated individuals) used by most of the contributors to our working group, it is perhaps not surprising that there was little concordance across contributions with respect to the genes identified as being associated with phenotype, apart from *MAP4*, which was consistently identified by those contributors who analyzed the simulated data. The simulation model was constructed such that *MAP4* contained variants with the strongest combined effects (accounting for 6.48% and 7.79% of the phenotypic variance in SBP and DBP, respectively), and as such, *MAP4* could be considered the easiest causal gene to find in the simulated data. Indeed, Ayers and Cordell [2014] showed that *MAP4* was clearly implicated even when a standard single-SNP analysis (rather than a gene-based approach) was used. Most findings in the real data did not reach significance thresholds that would withstand correction for multiple testing (of the numbers of genes investigated) or achieve compelling posterior probabilities of association. Therefore, the feeling in our working group was that most of even the top-ranking findings were most probably false positives or that they would at least require careful replication and validation in larger cohorts. Those contributors who analyzed the simulated data were able to formally examine type I error and power, either through examination of all 200 simulation replicates or through counting the numbers of true and false positives (i.e., estimating the proportion of truly or falsely associated genes that were detected) within a single replicate. Receiver operating curves generated by Liu et al. [2014] and Zhang et al. [2014] suggest that in most cases the power barely surpassed the type I error rate, a conclusion that was consistent with results presented by Yang et al. [2014], who found low power (3.8–14%) for achieving an uncorrected nominal *P*-value of 0.05.

The method most commonly used across the contributions was SKAT [Wu et al., 2011] or in some cases its extensions, SKAT-O [Lee et al., 2012] and famSKAT [Chen et al., 2013]. The popularity of SKAT appears to be largely due to its theoretical appeal and computational convenience. No clear advantage for SKAT in terms of power was seen from the investigations performed by our working group, although it is hard to make definitive statements in this regard, given the overall low power seen for any method when applied to the GAW18 data (particularly when analysis was restricted to unrelated individuals). However, Yang et al. [2014] did find SKAT to be sometimes superior to an alternative goodness-of-fit test.

## Conclusions

A variety of methods exist for constructing gene-based tests. Most approaches were found to be successful in detecting significant effects at *MAP4* in the GAW18 simulated data. No other genetic effects were consistently detected, most likely because of low power, particularly when analysis was restricted to a subset of unrelated individuals. This observation emphasizes the importance of the use and further development of analysis approaches that allow the inclusion of all individuals, including related individuals.
